# Accidental Deaths Due to Toxic Industrial Cyanide Inhalation: An Autopsy Case Report

**DOI:** 10.7759/cureus.25376

**Published:** 2022-05-26

**Authors:** Devendra Jadav, Ashish Saraf, Raghvendra S Shekhawat, Tanuj Kanchan, Aasma Nalwa

**Affiliations:** 1 Department of Forensic Medicine and Toxicology, All India Institute of Medical Sciences, Jodhpur, IND; 2 Department of Forensic Medicine and Toxicology, All India Institute of Medical Sciences, Gorakhpur, IND; 3 Department of Pathology & Lab Medicine, All India Institute of Medical Sciences, Jodhpur, IND

**Keywords:** personal protective equipment (ppe), industrial accidents, electroplating, chemical accident, forensic autopsy

## Abstract

Autopsies of accidental deaths in industrial scenarios have always been a challenging job for a forensic pathologist. Industries that employ chemical agents pose a unique risk, especially when safety protocols are ignored. Exposure to cyanide salts creates an additional risk since death may occur quickly. We present one such incident of the accidental deaths of three industrial workers, which could have been prevented if proper safety measures had been followed. Four workers fell unconscious while cleaning the electroplating chamber of the handicraft industry. Three were declared dead on arrival at the emergency department, while one survived. Autopsy of all three victims showed similar findings of pink-colored post-mortem staining and multiple petechial hemorrhages over the heart and lungs. After histopathological and chemical analysis, the cause of death was opined to be due to complications of cyanide poisoning. In accidental industrial deaths, the forensic pathologist should consider the possibility of death due to toxic chemicals, such as cyanide, used in the manufacturing process. The industrial personnel should be educated about the risks involved, and proper use of safety equipment should be encouraged to avoid such hazardous outcomes. Additionally, the people employed in the autopsy of the deaths related to chemical disasters should ensure their personal safety and preventive measures.

## Introduction

Investigation of accidental deaths in industrial scenarios has always been a challenging job for a forensic pathologist. Accidental deaths in industries can occur due to various reasons, viz., falls, electrocution, poisoning, etc. Sectors that employ chemical agents pose a unique risk to the workers, mostly when preventive and security protocols are ignored either by the employer or the employees due to various reasons. Hazardous xenobiotics present in the workplace can enter the body through ingestion, inhalation, or direct contact with the chemical. Inhalation of poison often results in quick death while raising suspicion of foul play. In this regard, cyanide salts, when inhaled, are known to result in a quick death.

Salts of cyanide have been described in the context of suicides, homicides, chemical warfare, iatrogenic exposure, and accidental episodes. Inadvertent episodes have occurred during household accidents, smoke inhalation in structural fires, in occupational and industrial disasters [1]. Industrial workers involved in photography, electroplating, chemical and plastic industries, polishing of gold and silver jewelry, etc., are at risk of cyanide poisoning as they use various salts of cyanide in the processing [2]. Diagnosis of acute cyanide poisoning is quite challenging as the healthcare workers are likely to miss its characteristic bitter almond odor and pinkish discoloration of the skin on physical examination [3,4]. We report an incident of fatal accidental cyanide poisoning in workers employed in an electroplating industry who succumbed to death quickly before reaching the emergency department.

## Case presentation

**Figure 1:**
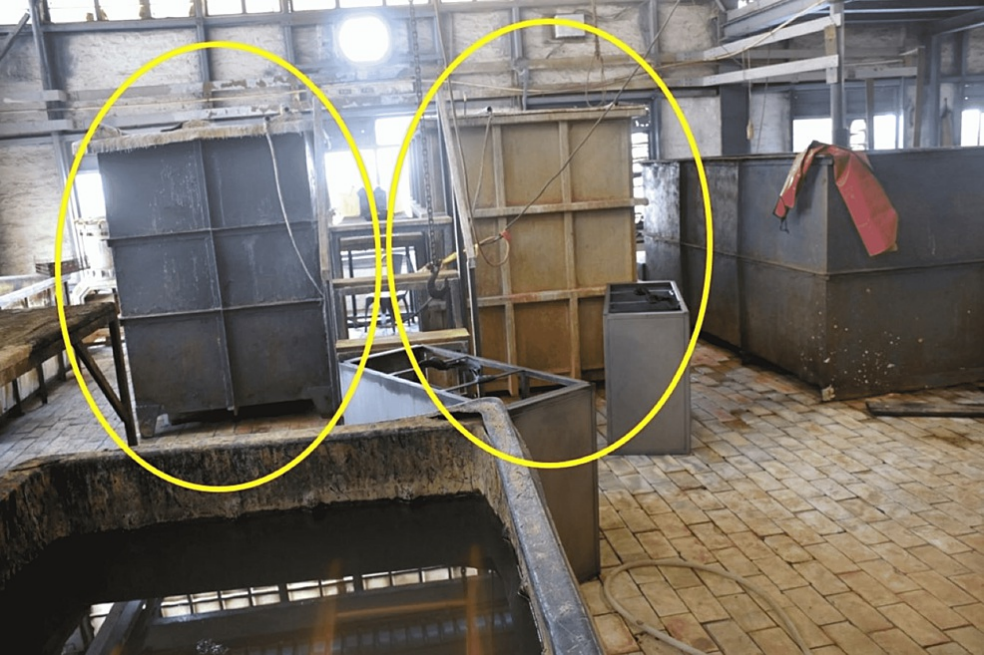
Scene of the incident where electroplating chambers were used in the process of electroplating (yellow circles)

The incident occurred in a handicraft industry in Jodhpur city, Rajasthan, India, where metallic furniture like office tables, chairs, racks, etc., are manufactured. As per the police information, four workers suddenly fell unconscious while working in the electroplating chamber. Three workers were declared dead on reaching the hospital, while one could be saved. Death scene investigation revealed that the industry had big electroplating tanks measuring about 10 feet × 6 feet × 3 feet in size, meant to do plating over the finished metallic furniture (Figure 1).

On the fateful day, two workers were cleaning the electroplating tank. One of them got into the tank, and another person helped him by giving him the cleaning material. Upon entering the tank, the first worker suddenly fell unconscious and did not reply to the second worker's call. The second worker entered the tank and fell unconscious too. The other two co-workers working nearby came to the rescue, and they also fell unconscious in the tank. All four were immediately transferred to a nearby hospital. Out of the four, three were declared dead when they were brought to the hospital, while one was admitted and later discharged healthy after two days.

**Figure 2:**
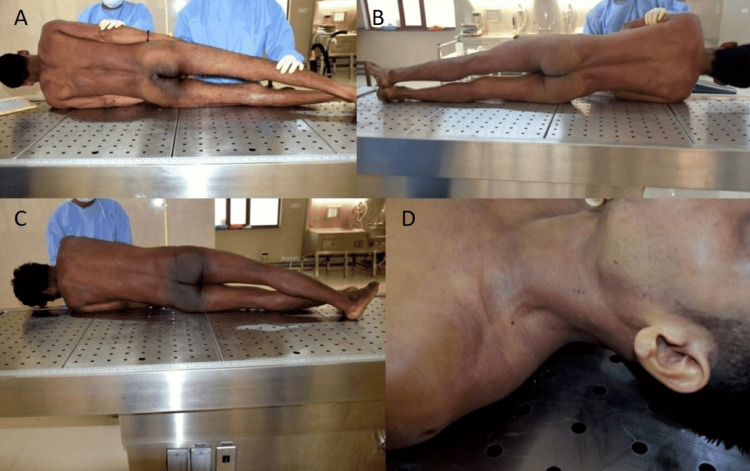
Pink colored post-mortem staining over the back of the body except in pressure areas in all three victims (A, B, and C) and over the left side of the neck and shoulder in one victim (D)

The police requested medico-legal autopsies for the three deceased workers. All the personnel participating in the autopsy wore personal protective equipment (PPE). At autopsy, all three deceased had similar findings. The bodies of all three deceased showed pinkish post-mortem staining over the back and dependent regions (Figure 2).

**Figure 3:**
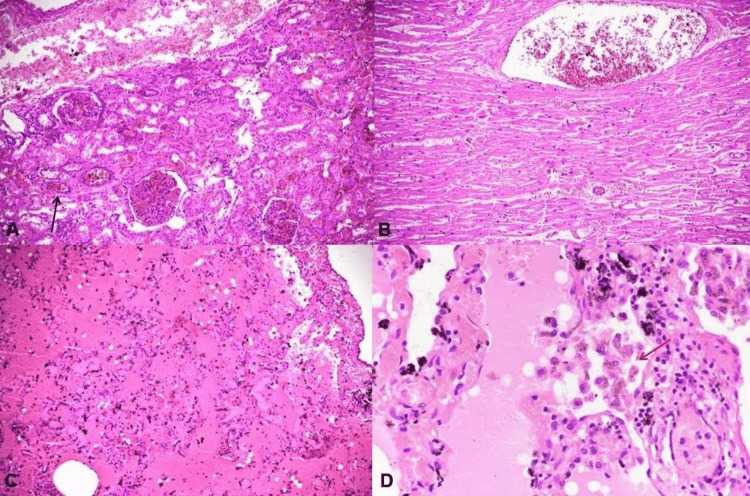
Histopathology of the organs A) Photomicrograph shows subcapsular hemorrhage in the kidney with underlying cortex showing congestion of glomerular capillary loops, blood-filled peritubular capillaries (black arrow), and acute tubular injury (H&E 100X). B) Myocardium shows fresh hemorrhage and congested capillaries (H&E 100X). C) Low-power view of the lung shows the diffuse accumulation of thin proteinaceous fluid in the alveoli with alveolar dilatation (H&E 100X). D) High power from the lung shows carbon pigment laden macrophages and heart failure cells (red arrow) in the alveoli, along with the presence of amorphous pink material (H&E 400X)

Internally, the mucosal wall of the respiratory tract was congested. Lungs were congested and oedematous. Both the lungs of all three deceased had multiple subpleural petechial hemorrhages. Petechial hemorrhages were also seen on the epicardium. The heart's walls, valves, and coronaries were intact without any significant diagnostic abnormality. The brain was congested and oedematous. The stomach mucosa was congested. The rest of the visceral organs were congested. The routine viscera, along with both lungs and clothes of all three deceased, were sent for chemical analysis, and the heart, lungs, spleen, liver, brain, and kidneys were subjected to histopathology. The lungs were sent with a paraffin oil coating to avoid evaporation of poison, if any. Histopathology of organs showed dilated alveolar spaces filled with proteinaceous fluid and congested blood vessels in the lungs, subcapsular hemorrhage with features of acute tubular injury in the kidneys, and fresh hemorrhages in the heart (Figure 3).

The toxicological analysis of viscera (blood and internal organs) and clothes gave positive tests for the presence of cyanide ions (CN­-), copper ions (Cu+2), zinc ions (Zn+2), ammonium ions (NH4+), nickel ions (Ni+2), chloride ions (Cl-), and sulfate ions (SO4-2), and gave negative test for other metallic poisons, ethyl, and methyl alcohol, alkaloids, barbiturates, tranquilizers, and insecticides. The cause of death was opined as complications of cyanide poisoning.

## Discussion

In 1782, the Swedish chemist Carl Wilhelm Scheele first isolated hydrogen cyanide from the Prussian blue pigment [4]. Hydrogen cyanide was used on the battlefield during World War I and later for genocide by Germans during World War II [5]. Potassium cyanide was used in the 1978 Jonestown mass suicide, in which more than 900 people died [6]. Cyanide has also been used for illegal euthanasia [7]. About industrial accidents, the biggest number of deaths was caused in 1984 in Bhopal, India, when over 5000 people died due to inhalation of methyl isocyanate and other cyanide chemicals [8]. Industries that either use or generate cyanide compounds during processing, including electroplating, metallurgy, leather tanning, photography, photoengraving, plastics manufacturing, and pesticide/ insecticide production, are at a major risk of accidental cyanide poisoning. Accidental exposure to cyanide salts increases the risk of toxicity for a significant number of people working in such places [8].

The electroplating process involves passing an electric current through a chemical solution to coat one metal object with another metal. The whole process consists of the use of hazardous chemicals like various metals, dissolvable salts of cyanide, sulfate and chromium, acid, and alkaline solutions during the entire process. The cyanide stabilizes the copper in the solution and allows for fine-grained co-deposition with tin at the same time. In solution, copper forms the complex with cyanide, which is relatively stable and has a good solubility. Free cyanide provides excellent anode corrosion in electrolytes with soluble anodes. Hydrogen cyanide contamination can occur either when the pH of a cyanide plating bath falls below ten (pH <10) or cyanide ions comes in contact with the acid. Possibilities of exposure to hazardous chemicals in an electroplating workplace can be associated with leakage or spillage of chemicals, inadequate ventilation, toxic fumes production, poor housekeeping, inadequate personal protective equipment (PPE), and accidental contact with contaminated PPE [9].

The electroplating sector in India is regulated by the Water Prevention & Control of Pollution Act 1974, the Air Prevention & Control of Pollution Act 1981, and the Hazardous Wastes Management, Handling, and Transboundary Movement Rules, 2008 [10]. The Central Pollution Control Board formed an expert committee in 2013 to look into the prospects of progressively eliminating cyanide from the electroplating process. The committee advised that cyanide be phased out of zinc and copper electroplating processes over three years [10]. In India, though, it is still utilized in the electroplating process of cadmium, gold, and silver.

In the reported cases, the possible cause for the formation of toxic cyanide gas is the contact of electrolyte solutions containing cyanide ions with the acid while cleaning the electroplating tank, and the possible cause for exposure to such toxic gas is the unavailability of PPE. Sadhra et al. conducted a study on electroplaters in West Midlands, England, to compare the responses of workers and safety experts regarding the safety measures and concluded that workers consider PPE as the most effective safety measure, while experts gave it the least importance [11]. Many cyanide-free alternatives are being investigated in the process of electroplating to avoid the risk associated with the use of cyanide [12].

The onset of cyanide poisoning symptoms is reported within seconds on inhalation or intravenous injection of water-soluble cyanide salts and takes several minutes to appear on ingestion of inorganic cyanide salts [4]. The toxic effects of cyanide are mainly due to the inhibition of aerobic respiration and initiation of anaerobic respiration at the cellular level [13]. Early toxic effects are mainly neurological, including dizziness, headache, nausea, and anxiety, which progresses toward severe harmful effects, including coma, seizure, respiratory depression, hypotension, and tachycardia. The triad of severe cyanide toxicity includes hypotension, altered mental status, and lactic acidosis [14]. Central respiratory arrest due to histotoxic hypoxia of the respiratory center is the most attributed mode of death in cyanide toxicity [15]. The final cardiorespiratory arrest occurs within 15 minutes of inhalation, as observed in the present cases [16].

In suspected inhalation cyanide exposure, the patient should first be evacuated from the contaminated area. During the evacuation, protective measures like face masks, eye shields, and double gloves should be used. Gloves should be changed frequently; alternatively, butyl rubber gloves can be used [17]. In such cases, 100% oxygen should be administered as soon as possible as cyanide causes decreased oxygen utilization. The use of such oxygen to compete with cyanide for the cytochrome oxidase binding sites may enhance the effect of antidotal therapy [18]. The antidotal kit for cyanide contains amyl nitrite, sodium nitrite, and sodium thiosulfate. However, due to the side effects of nitrites, the combination of hydroxocobalamin and thiosulphate is considered a better alternative for the treatment of cyanide poisoning [19]. European countries use 4-dimethylaminoethano(4-DMAP) as a methemoglobin inducer of choice [1]. Morningstar et al. demonstrated the use of intramuscular administration of hexachloroplatinate (HCP) as an effective antidote for a lethal dose of cyanide in rabbits [20].

## Conclusions

In accidental industrial deaths, the possibility of death due to toxic chemicals like cyanide should be kept in mind. In developing countries, workers handle these toxic compounds without wearing the required protective equipment, which leads to increased morbidity and mortality. They need to be provided with protective gear to protect them from the harmful effect of chemicals. Even if the PPE is available, workers often do not use it, so their sensitization is vital along with law enforcement. The use of cyanide-free solutions in the electroplating industries should be investigated and encouraged due to the toxic effects of cyanide. Additionally, the people employed in the autopsy of the deaths related to chemical disasters should ensure their safety and preventive measures.
